# Dual-Task Treadmill Training for the Prevention of Falls in Parkinson's Disease: Rationale and Study Design

**DOI:** 10.3389/fresc.2021.774658

**Published:** 2022-03-02

**Authors:** Veit Mylius, Laura Maes, Katrin Negele, Christine Schmid, Ramona Sylvester, Caroline Sharon Brook, Florian Brugger, Santiago Perez-Lloret, Jens Bansi, Kamiar Aminian, Anisoara Paraschiv-Ionescu, Roman Gonzenbach, Peter Brugger

**Affiliations:** ^1^Department of Neurology, Center for Neurorehabilitation, Valens, Switzerland; ^2^Department of Neurology, Philipps University, Marburg, Germany; ^3^Department of Neurology, Kantonsspital St. Gallen, St. Gallen, Switzerland; ^4^Biomedical Research Center (CAECIHS-UAI), National Research Council (CONICET), Buenos Aires, Argentina; ^5^Facultad de Medicina, Pontificia Universidad Católica Argentina, Buenos Aires, Argentina; ^6^Departamento de Fisiología, Facultad de Medicina, Universidad de Buenos Aires, Buenos Aires, Argentina; ^7^Department of Health, Physiotherapy, OST–Eastern Swiss University of Applied Sciences, St. Gallen, Switzerland; ^8^Laboratory of Movement Analysis and Measurement, EPFL, Lausanne, Switzerland; ^9^Department of Psychiatry, University of Zurich, Zurich, Switzerland

**Keywords:** dual tasking, augmented reality, treadmill, Parkinson's disease, random number generation, executive function

## Abstract

Various factors, such as fear of falling, postural instability, and altered executive function, contribute to the high risk of falling in Parkinson's disease (PD). Dual-task training is an established method to reduce this risk. Motor-perceptual task combinations typically require a patient to walk while simultaneously engaging in a perceptual task. Motor-executive dual-tasking (DT) combines locomotion with executive function tasks. One augmented reality treadmill training (AR-TT) study revealed promising results of a perceptual dual-task training with a markedly reduced frequency of falls especially in patients with PD. We here propose to compare the effects of two types of concurrent tasks, perceptual and executive, on high-intensity TT). Patients will be trained with TT alone, in combination with an augmented reality *perceptual* DT (AR-TT) or with an *executive* DT (Random Number Generation; RNG-TT). The results are expected to inform research on therapeutic strategies for the training of balance in PD.

## Introduction

Falls occur with a high prevalence of about 60% in patients with Parkinson's disease (PD) with 40% having recurrent falls with an average of 20 falls per year (1). Stooped posture, increased sway, turning deficits, slowed integration of somatosensory information, reduced postural, and anticipatory adjustments are important factors contributing to reduced balance (2). Already in an early stage of PD, diminished gait speed, pain, history of falls or near falls, balance disturbances during dual-tasking (DT), retropulsion, freezing of gait (FoG), and the need for stabilization assistance are associated with falls (3). When controlled for age and sex, only fear of falling, history of near falls, and retropulsion predict prospective falls (3). In later stages, the akinetic-rigid motor type, motor fluctuations (off-phases and dyskinesia), high L-dopa dosage, FoG, and anxiety predict falls (4, 5). At these stages, falls are further predicted by impaired postural stability and by impaired cognitive function. In particular, attention, executive function, and specifically the capability of DT predict future falls (6, 7).

Two hypotheses for DT deficits may explain the reduced performance in patients with PD (8). The capacity hypothesis assumes limited frontal-executive resources (9), while the bottleneck hypothesis assumes that the time factor leads to a reduced availability for two different tasks to be performed simultaneously (10). The two views are not mutually exclusive. On the one hand, diminished executive functions may hamper the capacity for motor execution while slowness, especially during dopamine depletion (off-phase), may simply delay motor execution. Another key factor to be considered is reduced automaticity, which requires a compensatory frontal-executive activation as shown in an interventional neuroimaging study (11). In view of reduced automaticity in postural reactions, physiotherapeutic interventions aim at employing goal-directed reward-based training enhancing both motor and cognitive neuroplasticity (12). DT is a widely used methodology for the quantification of performance decrements induced by well-defined task-load (8). In patients with PD, DT reduces step width and step variability already during the early stages (13) presumably due to a wrong prioritization of the cognitive task, which then leads to a deterioration of gait and posture (14). The nature of the dual-task seems to be critical. When “pure motor” and “motor-cognitive” DT were compared, especially motor DT deficits were found to be a predictor of falls (15).

Since physiotherapeutic interventions often failed to report substantial influences on gait stability (16), goal- and reward-based dual-task treadmill training in augmented reality (AR-TT) has been developed (17). By simulating everyday situations, it reduced the frequency of falls as compared to TT alone in elderly persons and patients with PD by training of dual-task capabilities, such as perceptual cueing (17). Neuroimaging analyses revealed diminished frontal but augmented cerebellar activity presumably contributing to increased gait stability following AR-TT with perceptual cues (11). Thus, both motor-perceptual and motor-executive DT appear to be powerful interventions in the training of gait.

Recent AR-TT solutions provide a range of different tasks suitable for PD patients with gait instability, which have to be chosen from the assessors to provide an optimal training adapted to the individual patient. From our experiences and from previous studies, dual-task interventions on the display that include reward games and cueing interventions on the floor are thought to have the greatest impact on gait in PD (17). Since both executive DT and perceptual DT contribute to improved gait performance, we aim at comparing pure TT with a TT combined with the perceptual challenge of AR-TT on the one hand and paired with a simultaneous executive function task on the other hand.

Several reasons made us select random number generation (RNG) as the executive task to be performed simultaneously with walking (RNG-TT). First, ever since Baddeley (18), this task has widely been used in the dual-task literature [see Dirnberger and Jahanshahi (19) for a review], especially also in patients with PD. In this latter context, Brown et al. (20) found that patients reacted opposite to controls when RNG was added to a motor task (manual tracking). Specifically, patients with PD showed an exacerbated bias compared to single-task conditions. The authors concluded that RNG is a useful tool for investigating executive functions, also in PD.

Second, dual-task experiments in patients with PD have previously helped to uncover factors leading to impairments in both cognition and motor action (20). Third, the impact of RNG on treadmill walking has recently been studied by Rodrigues et al. (21), albeit not in patients with PD, but in a group of participants aged 60–85 years. Forth, RNG is a highly demanding task, yet it is playful (there are no “correct” or “incorrect” responses), relatively independent of education (22), and resistant to multiple testing (23). Finally, one advantage of RNG as a dual-task is that performance quality can be exactly quantified. This means that the bidirectional interference (24) between the two tasks performed simultaneously can be fully described (e.g., RNG can reduce step length or impair other measurable gait parameters, but at the same time walking could enhance sequence redundancy or impair other measures of sequential randomness).

## Materials and Methods

### Study Design

**Figure 1:**
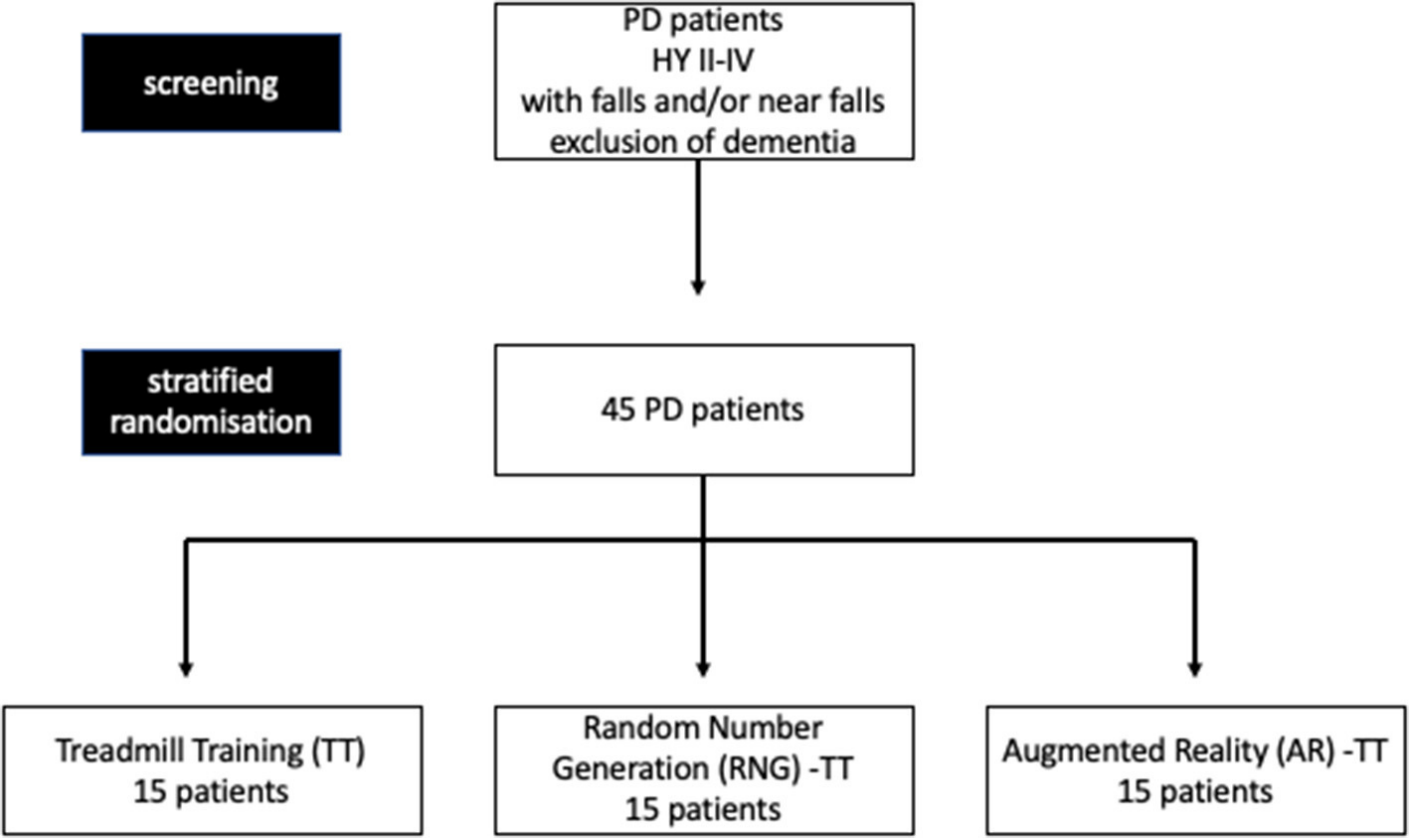
Flowchart of the study.

**Figure 2:**
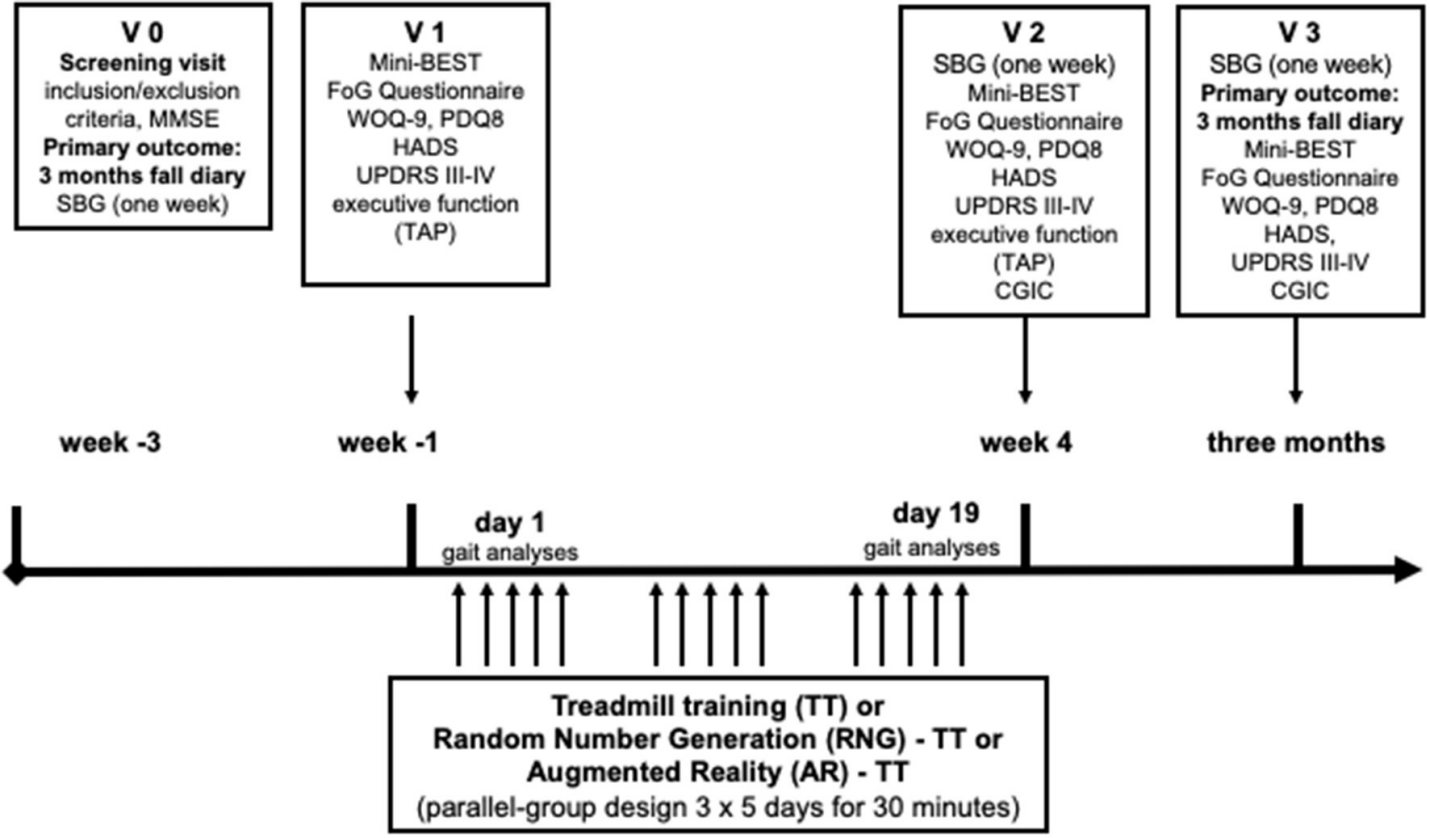
Experimental protocol design. MMSE, Mini-Mental Status Examination; Mini-BEST, Mini-Balance Evaluation Systems Test; SBG, sensor-based gait analyses; FoG, freezing of gait; WOQ-9, Wearing-Off Questionnaire-9; PDQ8, QoL in PD questionnaire; HADS, Hospital Anxiety and Depression Questionnaire; MDS-UPDRS, MDS-sponsored revision of the Unified Parkinson's Disease Rating Scale; CGIC, Clinical Global Impression of Change; TAP, Test Battery of Attentional Performance.

Patients with PD will be assigned randomly to one of three different training programs (AR-TT, or RNG- TT, or TT alone), which will be performed in a parallel-group design for 3 weeks of 30 min daily for 5 days a week in addition to the regular rehabilitation program (Figures 1, 2). Frequency of falls or near falls (within 3 months before V1) will be used (x > 1 or x = 1) for stratified randomization.

Assessments will be performed by a blinded investigator before the beginning of the training (V1), after 3 weeks of training (V2), and 3 months after training (V3) during the on-phase. The primary outcome will be the frequency of falls or near-falls during 3 months following training compared to the frequency of falls or near-falls in the 3 months before inclusion (AR-TT vs. TT, and RNG-TT vs. TT). Both interventions will be compared in exploratory analyses (AR-TT and RNG-TT).

Secondary outcomes will be the clinical parameters Movement Disorder Society-sponsored revision of the Unified Parkinson's Disease Rating Scale (MDS-UPDRS III-IV) (25), Hospital Anxiety and Depression Questionnaire (HADS) (26), FoG Questionnaire (27), the Mini-Balance Evaluation Systems Test (Mini-BEST) (28), quality of life in PD questionnaire (PDQ-8) (29), Wearing-Off Questionnaire-9 (WOQ-9) (30), and executive function testing by using tests of the Test Battery of Attentional Performance (TAP) (31) assessed at V1, and compared to V2 and V3 (MDS-UPDRS III-IV, HADS, FoG Questionnaire, Mini-BEST only). Clinical Global Impression of Change (CGIC) will be assessed and V2 and V3 (32).

Sensor-based gait (SBG) assessment will be measured and compared before V1, after V2, and before V3 to have more detailed information on gait changes in the natural environment at home. Given the set of parameters described in Section “Gait and Physical Activity Assessment Using Wearable Sensors,” analyses will be conducted to evaluate the sensitivity to change related to a progression in daily-life motor performance following the rehabilitation program. In addition, gait analysis on behalf of the C-Mill data will be collected before and after the intervention during 3 min of comfortable walking (V1 and V2).

### Patients

In total, 45 PD patients (age 40 or older) with Hoehn and Yahr stages II-IV (only mobile patients with advanced PD), with at least one fall or near-fall within the 3 months before baseline assessment, will be asked for participation when appointed to in-ward neurorehabilitation at the Center for Neurorehabilitation in Valens, Switzerland (Figure 1). Patients shall be able to participate in the treadmill training at high intensity for 3 weeks. Patients with dementia, defined by a Mini-Mental Status Examination (MMSE) test result of <24, will be excluded from participation. The protocol was approved by Ethikkommission Ostschweiz (BASEC ID 2019-01894) and registered at ClinicalTrials.gov (Identifier: NCT04108741).

### Experimental Intervention

**Figure 3:**
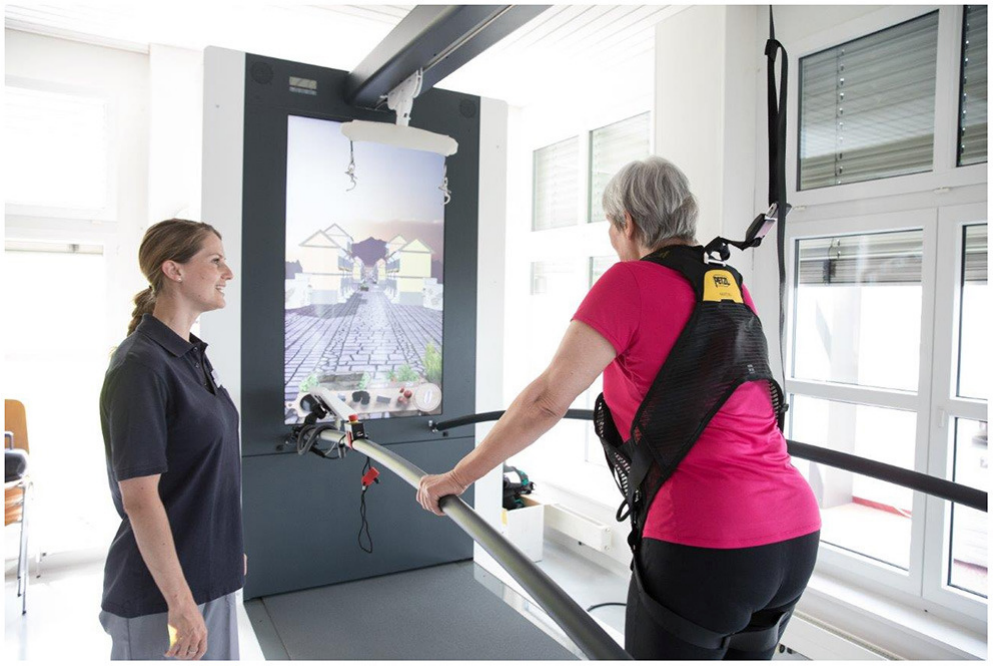
Treadmill training by using the C-Mill (MOTEK, the Netherlands) which may include training on a display and on the floor (augmented reality).

All three arms of the study will get their usual in-ward rehabilitation with additional treadmill sessions 5 times weekly for 3 weeks. The three treadmill sessions vary only with respect to the AR and the RNG component added to TT alone. The three groups will be trained at 70% of the maximal convenient velocity of each patient to train with high intensity as suggested (33). A recent meta-analysis showed that high-intensive training (HIT) is feasible and safe in persons with PD (34). The authors concluded that there is clear and strong evidence for resistance training to improve muscle strength and moderate evidence for endurance training to improve cardiorespiratory fitness. The speed will be increased stepwise according to baseline measurements. For TT, we will employ the AR-based treadmill from Motek (C-Mill, Motek Medical B.V., The Netherlands, Figure 3).

In the AR group, different mixed programs (based on the standard software) adapted to the need of PD patients with balance deficits were designed for each week to increase difficulty (e.g., stepping stones, speeding-up) for 30 min for 5 days a week for 3 weeks. Stepping stones consist of virtual barriers displayed on the treadmill ground to train external cueing. In the subprogram “speeding-up,” patients can change their velocity according to signs on the floor to train their walking flexibility. At the end of the treadmill training, we selected a longer period from 5 to 6 min with a complex dual-task paradigm with increased difficulty in each week. In the first week, we will employ the “Italian Alps.” Italian Alps consists of an AR with a DT displayed on a display in front of the patients. While walking, the patient move to collect the ingredients for a pizza displayed on the way on different sides. These ingredients can be reached by moving sideward but only necessary ingredients shall be taken as displayed on the screen. In the second week, we will use “Arcanoid.” This is a balance game on the display in which the patients have to reduce the geometric figures at the top by moving a ball that hits a plate on the bottom that can be moved by their feet. In the third week, we will administer “Monster Games.” In Monster Games, the patients have to decide whether to pick up a reward or to avoid a monster on the floor of the treadmill. Most games give a visual feedback either on the display or on the treadmill floor. There is also an auditory feedback provided in some applications but turned off for the present investigation.

In the RNG-TT group, we employ TT as described above but with RNG as the simultaneous secondary task. RNG will be performed every second day during treadmill training (4 blocks of 100 s each, separated by 4-min blocks of pure walking). Specifically, the Mental Dice Task (35, 36) will be administered. During 100 s, patients have to produce the digits of a single die in a sequence as random as possible. The generation will be individually paced, i.e., in a rhythm provided by the patient's gait, such that one number for every right-foot placement will be to be generated. Number generation will be tape-recorded, and the sequence will be later analyzed to characterize its information-theoretic properties (omitted steps and “rule breaks,” i.e., digits like 7, will also be recorded). This will allow quantification of the interference between walking and number generation. Every second session will be administered under dual-task conditions.

In the TT group, we will also employ the C-Mill treadmill, but use the display only for walking purposes without any tasks. Walking speed will be increased stepwise in analogy to the other interventions.

### Gait and Physical Activity Assessment Using Wearable Sensors

Monitoring using wearable inertial devices [SBG analyses that includes 3-axial accelerometers and gyroscopes (Physiolog5^®^, Gait Up, Switzerland)] will allow an objective quantification of motor activity in daily life, during the course of the study three times for 1 week during the daytime (before and after 3 weeks of training and after 3 months following the intervention; Figure 2). Synchronized inertial devices will be attached to both feet (two sensors fixed by a clip on the shoes or at the ankle with a tape) and at the lower back (one sensor fixed with a double-sided adhesive tape and secured with a band-aid at the area of the fourth and fifth lumbar vertebrae) to record accelerations and angular velocity of the respective body segments. The study nurse and an instruction sheet will explain the use of the sensors at home for the week before the first visit (V1). The sensors for the week after V2 and the week before V3 will either be sent and/or collected by regular mail or given and/or collected during the appointments. A protocol with four columns (FoG, sports and therapy, weather and activities, and particularity) will be given to the patient. Recorded data will be transferred *via* online sharing to the Laboratory of Movement Analysis and Measurement, École Polytechnique Fédérale de Lausanne (EPFL), Lausanne, Switzerland. It will be processed using validated state-of-the-art algorithms (37–41) to characterize the multiple dimensions of gait and physical activity. More specifically, the parameters extracted will be related to.

#### Ambulatory Activity and Walking Behavior

Step count [number per day], distribution of walking bout duration [seconds], the timing of walking activity over the course of the day [% of time spent walking in the morning vs. afternoon], and indicators of walking performance, expressed, for example, as the percentage of long walking bouts with fast and regular speed [%] (42).

#### Gait Pattern

Spatio-temporal gait parameters, such as speed [m/s], cadence [steps/min], stride length [m], swing/stance/double support phases [% of gait cycle duration], variability [unitless], and asymmetry [unitless].

#### Turning

Automatic detection of turning and estimation of gait parameters, step count, and movement smoothness during the turning period. Detailed characterization of turning is expected to provide further insights into postural instability and fall prediction, as it requires multi-limb coordination and continuous displacement of the body center of mass, and it is regularly performed during daily activities [it is estimated that up to 50% of strides taken during daily activities are turning strides (43)]. On top of being a significant motor parameter of gait performance, turning behavior is an important dopamine-mediated predictor of neuropsychiatric symptoms [e.g., (44, 45)]. Novel therapies focusing on turning can improve clinical outcomes in PD, such as the frequency of falls (46).

#### Freezing of Gait

Combined inertial data from feet and lower back sensors will be used to extract a set of features that will be used to identify FoG episodes using machine learning-based algorithms (47), previously developed and validated on similar datasets (48, 49).

All the analyses will be performed by processing the raw sensor data [i.e., acceleration [gravity units, g] and angular velocity (degrees/s)] using algorithms implemented in MATLAB (MathWorks Inc, Portola Valley, CA, USA).

### Gait Analyses of the C-Mill Data

Three minutes of walking with the comfortable velocity will be used for gait analyses at V1 and V2 (Figure 2) (at the same velocity as at V1). The following parameters will be determined: velocity [m/s], distance [m], steps [number], and step frequency [number/time unit]. Step length [m], stand time [min], and step width [m] will be calculated for both feet separately. In addition, the raw data will be processed by using MATLAB (MathWorks Inc, Portola Valley, CA, USA).

### Sample Size

According to previous studies, a reduction of 50% in the frequency of falls or near falls can be expected in the two experimental intervention groups vs. a reduction of 25% in the control group. Under the assumption of a difference of 1.5 falls between the respective group and placebo and an SD of 1.16, a sample size of 15 patients in each group is required (alpha = 0.025 two-tailed, power = 80%), i.e., 10% dropouts (G-power) (11).

### Statistics

Numerical variables will be described with means and SDs, and categorical variables by percentages. The numerical outcomes in this study will be analyzed by a mixed-effect ANOVA model, using treatment and visit as fixed factors, subject as a random factor, and baseline values as covariates. A planned contrast will be used to compare data at month 3 between the groups. Non-normally distributed variables will be log-transformed. Imbalances in the characteristics of the groups at baseline will be included in the ANOVA model as part of a sensitivity analysis. The statistical analysis will be conducted in R 4.1.1 (The R Foundation, Vienna, Austria).

## Results

In a randomized-controlled study with a parallel-group design, one of the three interventions will be applied over 3 weeks during regular neurorehabilitation with a follow-up of three months (AR-TT vs. RNG-TT vs. TT). Cognitive executive performance, anxiety, FoG, motor function, gait analyses, and functional gait testing will be controlled. In addition, wearable sensors will allow for the quantification of gait performance during daily living.

We expect the participation of 45 patients with a similar distribution of falls and further baseline factors in the three intervention arms. We assume that dual-task training with both executive and perceptual concurrent tasks leads to better gait performance than TT alone. Secondary outcome measures were chosen to elucidate influencing factors (executive function, mood, and motor function) and to show training effects on further outcomes (i.e., gait speed and clinical gait testing). We hope that SBG analyses will reveal which intervention has more impact on improving gait stability, on FoG, and on gait performance in daily life.

## Discussion

This study examines the effects of dual-task training added to HIT TT on falls and gait in PD. It assesses whether motor-perceptual dual-task training by means of AR (AR-TT) and a highly demanding motor-executive DT, i.e., the generation of a random number at every right foot placement (RNG-TT), can realize further improvements over TT alone.

Already TT, especially at high intensity, has a marked influence on the reduction of falls (17, 33). The additional interventions use different strategies (perceptual or executive) to increase neuroplasticity. Both shall further increase the ability to react toward sudden obstacles by training either perceptual or executive DT. The previous AR-TT study revealed reduced falls as compared to TT during a follow-up of 6 months (17) presumably due to a reduced cognitive frontal load (i.e., more capacity for executive reactions) and due to an increased cerebellar motor control (11). This study aims at enhancing the effects of AR-TT on gait in a shorter but more intense training period by using programs adapted for PD patients with a balance disorder. In addition, in a third arm, RNG will be probed as an executive-cognitive task to further improve baseline TT.

Which of the additional training paradigms will have more advantages compared to TT alone will be elucidated in the present study. Previous investigations revealed that cognitive training in PD should have a game-like character comparable to commercially available games [Nintendo –wii; (50)]. We assume that the game-like character of RNG may exhibit a powerful effect on the ability of a patient for DT. In fact, RNG studies demonstrated that performance decrements on the simultaneous motor task are considerable, even in healthy subjects. Diverse kinds of motor tasks were investigated, from simple finger tapping (51), key pressing (52), and mouse clicking (24) to grooved pegboard performance (53), pursuit tracking (54) and bimanual coordination (55). Apart from the study cited in the introduction section (21), walking was not specifically investigated, but other complex motor coordination tasks, such as car driving (56), to which RNG introduced more jerky actions. For the influence of RNG on the performance of patients with PD on other cognitive tasks see Witt et al. (57) and Robertson et al. (58). To the (pronounced) degree that RNG interferes with motor execution, we would expect the training of DT based on RNG during locomotion also to lead to a pronounced improvement in everyday gait performance. On the other hand, AR-TT has various advantages compared to executive DT. The game-like character and the diversity of different motor tasks are important features to enhance cognitive and motor neuroplasticity in patients with PD suffering from reduced and slowed motor reactions.

The study further assesses the conditions and influencing factors underlying the training effects in PD, which have not been studied in the previous investigation focusing on healthy elderly with falls (17). We finally plan to use the most recent AR-TT and adapt the AR programs for PD patients with balance deficits by including cueing and perceptual DT.

In summary, the interventions were designed to increase gait automaticity and to compensate for any reduced executive functioning in the course of the disease. Whethermotor-perceptual DT has stronger effects than motor-executive DT or whether the contrary will be the case, cannot be answered so far. The present study may well provide an answer.

### Limitations

Not all factors influencing gait stability can be controlled. We aim at maintaining dopaminergic stimulation unchanged, but fluctuations of the disease, and of concomitant medication remain an influencing factor. Since the respective dopaminergic state (which can hardly be controlled for) influences some of the outcome measures, we will employ SBG analyses allowing for gait assessment during daily life. In addition, we are planning to assess wearing-off and motor fluctuations. Influencing factors, such as orthostatic hypotension, concomitant diseases, medication, and polyneuropathy, will be recorded and considered. Moreover, it is difficult to match the motor-perceptual and the motor-executive DTs with respect to task difficulty. But also in this respect, the planned study will generate data from which further improvement in the rehabilitation of gait impairment and QoL in PD can be expected.

## Data Availability Statement

The original contributions presented in the study are included in the article/supplementary material. Further inquiries can be directed to the corresponding author.

## Author Contributions

VM and PB contributed to the design, financing and execution of the study, and writing of the first draft and corrections. SP-L contributed to the statistics section. LM, KN, CS, RS, CB, FB, JB, and RG contributed to the design and the execution of the study. KA and AP-I contributed to the sections on sensors. All authors reviewed the final version of the manuscript and gave their approval.

## Conflict of Interest

The authors declare that the research was conducted in the absence of any commercial or financial relationships that could be construed as a potential conflict of interest.

## Publisher's Note

All claims expressed in this article are solely those of the authors and do not necessarily represent those of their affiliated organizations, or those of the publisher, the editors and the reviewers. Any product that may be evaluated in this article, or claim that may be made by its manufacturer, is not guaranteed or endorsed by the publisher.
